# Comparison between repeat anterior and posterior decompression and fusion in the treatment of two-level symptomatic adjacent segment disease after anterior cervical arthrodesis

**DOI:** 10.1186/s13018-020-01834-z

**Published:** 2020-08-08

**Authors:** Junming Cao, Can Qi, Yipeng Yang, Tao Lei, Linfeng Wang, Yong Shen

**Affiliations:** Department of Orthopedics, The Third Hospital of Hebei Medical University, The Key Laboratory of Orthopedic Biomechanics of Hebei Province, 139 Ziqiang Road, Shijiazhuang, 050051 China

**Keywords:** Anterior discectomy and fusion, Adjacent segment disease, Two levels, Posterior, Revision surgery

## Abstract

**Background:**

Two-level symptomatic adjacent segment disease (ASD) is rarely reported, but remains a challenge after anterior cervical arthrodesis. The purpose of this study was to compare the clinical and radiological outcomes of repeat anterior and posterior decompression and fusion procedures for two-level symptomatic ASD.

**Methods:**

Thirty-two patients with two-level symptomatic ASD were retrospectively reviewed and underwent repeat anterior cervical discectomy and fusion (ACDF) or posterior decompression and fusion (PDF). Clinical outcomes (JOA, NDI, and VAS scores), perioperative parameters (blood loss, operation time, and length of hospital stay), radiological parameters (cervical lordosis and ROM), and complications were compared.

**Results:**

Eighteen patients underwent ACDF, and 14 patients underwent PDF. Patients who underwent PDF were older, more frequently presented with myelopathic deficits, and were fused at more levels. Patients who underwent ACDF experienced significantly shorter surgery time (*p* < 0.001), lower blood loss (*p* < 0.001), and reduced hospital stay (*p* = 0.002). Both groups exhibited significant increases in JOA scores and decreases in NDI and both neck pain and arm pain VAS scores (*p* < 0.05), but patients who underwent PDF had significantly higher NDI scores (*p* = 0.012), neck pain VAS scores (*p* = 0.019), loss of cervical lordosis (*p* < 0.001), and loss of ROM (*p* = 0.001). Three patients developed dysphagia in the ACDF group, and two patients had C5 root palsy and one had hematoma in the PDF group. Recurrent ASD after the second operation occurred in two patients in the ACDF group but no patients in the PDF group.

**Conclusions:**

For patients with two-level symptomatic ASD, both anterior and posterior decompression and fusion were effective for improving the neurological function. For patients with radicular symptoms, ACDF had less surgical trauma, better restoration of lordosis, and less postoperative neck pain, but higher chance of recurrent ASD. PDF was an effective surgical option for older patients with myelopathy developing in adjacent segments.

## Background

Anterior cervical decompression (discectomy or corpectomy) and fusion (ACDF or ACCF, respectively) has been the gold standard treatment for degenerative cervical spine diseases [1, 2]. However, adjacent segment disease (ASD), defined as new radicular or myelopathic symptoms and new imaging evidence of degenerative changes at levels adjacent to the previous arthrodesis [3, 4], is one of the main problems associated with anterior cervical arthrodesis. Regardless of whether ASD represents enhanced degeneration due to adjacent fusions or merely natural progression of degeneration [5, 6], patients with invalid conservative treatment require further surgical treatment. Revision surgery for symptomatic ASD after ACDF was reported to be necessary in 5.1% to 22.2% of cases [3, 4, 7–9].

According to the levels affected, ASD can be found in the superior, inferior, or both adjacent levels. Single-level ASD can be treated by second anterior fusion [7, 10, 11], laminoplasty [12], or artificial disc replacement [13, 14]. Meanwhile, two-level ASD, defined as the development of new neurological symptoms in both cranial and caudal levels or two contiguous levels in directly adjacent discs, is less common [7, 8, 11], but represents a great challenge for surgeons. There are limited data regarding the revision approaches and clinical outcomes for two-level ASD. The purpose of this study was to elucidate the effectiveness of repeat anterior and posterior decompression and fusion for two-level symptomatic ASD by reviewing the surgical and radiological outcomes.

## Materials and methods

After obtaining Institutional Review Board approval and in accordance with the STROBE statement, we retrospectively reviewed the records of patients who underwent revision surgery for two-level symptomatic ASD at a single institution between January 2006 and January 2016. The inclusion criteria were (1) symptomatic ASD, defined as patients who underwent initial ACDF or ACCF and developed new radicular or myelopathic symptoms, (2) responsible lesions at both cranial and caudal levels or two contiguous levels directly adjacent to the previous arthrodesis confirmed by magnetic resonance imaging (MRI) and physical examination, and (3) no response to conservative treatment for at least 6 weeks and receipt of revision surgery. Patients with cervical spine traumas, tumor spinal pathologies, neoplasms, spinal infections, congenital deformations, and chronic systemic illnesses were excluded.

The patients comprised 19 men and 13 women, with a mean age of 53 years (range, 39 to 75 years). The mean period from primary operation to revision operation was 6.5 years (range, 2.6 to 10.7 years). Symptoms before revision surgery included myelopathic symptoms (*n* = 11), radicular symptoms (*n* = 13), or both types of symptoms (*n* = 8). Twenty patients underwent initial ACDF with a polyetheretherketone (PEEK) cage and traditional plate implantation, and 12 patients underwent initial ACCF with a titanium mesh or autogenous iliac bone graft and instrumentation. The previous arthrodesis was performed at our hospital in 22 patients and at other hospitals in 10 patients. The primary fused levels and adjacent pathological levels are shown in Table 1.

**Table 1:** Description of primary and revision levels

Primary procedure and fused levels	Adjacent segment and reoperation level	Number (*N* = 32)
ACDF in C4/5	C3/4, C5/6	5
ACDF in C5/6	C4/5, C6/7	5
ACDF in C5/6	C3/4, C4/5	4
ACDF in C4/5	C5/6, C6/7	6
ACCF in C5	C3/4, C6/7	6
ACCF in C5	C6/7, C7/T1	1
ACCF in C4	C5/6, C6/7	4
ACCF in C6	C3/4, C4/5	1

According to the surgical approach, the patients were divided into two groups: the ACDF group and the posterior decompression and fusion (PDF) group. The quality (soft or ossified) and position (disc level or retrovertebral) of compression, presenting symptoms, and pathological extent were the main factors considered during selection of the surgical approaches. The indications for repeat ACDF were (1) disc herniation without osteophyte mainly located just behind a disc and (2) primary manifestation as radiculopathy or mild myelopathy without spinal canal stenosis that could be effectively decompressed by anterior discectomy. The indications for repeat PDF were (1) prolapsed intervertebral disc behind the vertebral body, (2) primary manifestation as myelopathy especially with intramedullary increased signal intensity (ISI) on T2-weighted imaging, (3) large osteophyte or ossification of the posterior longitudinal ligament (OPLL), (4) narrow spinal canal at the primary operative segments that required decompression through a posterior approach, and (5) cervicothoracic disease.

### Surgical technique

**Fig. 1:**
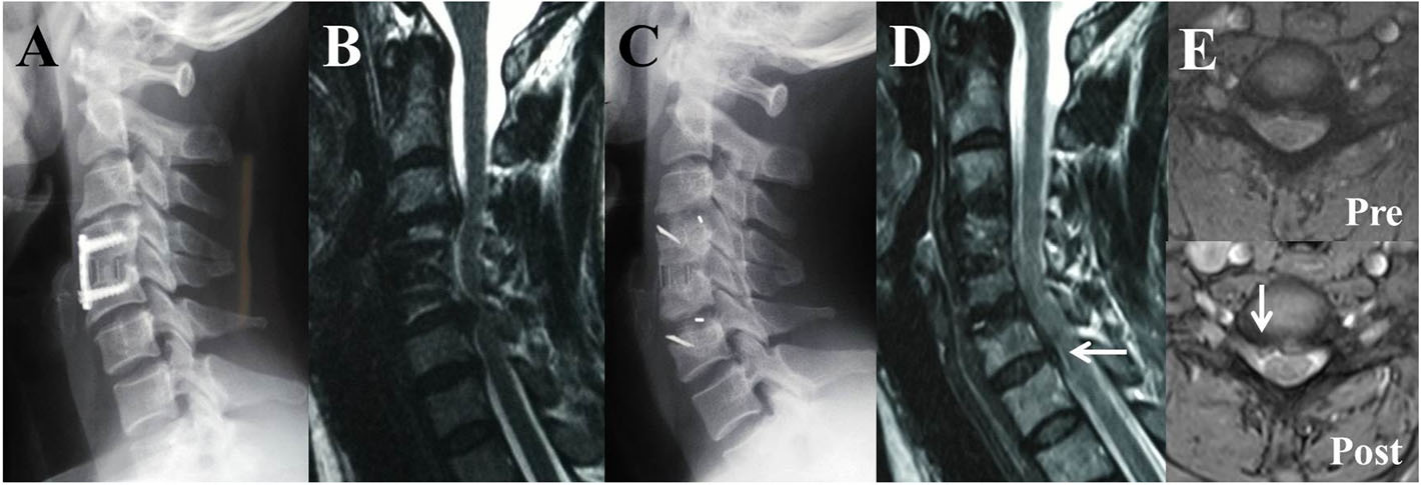
A 41-year-old woman developed two-level ASD with radicular symptoms at the 5th year after the primary surgery and underwent ACDF with two Zero-P cages as a revision surgery. **a** Lateral radiograph after the primary surgery showing that ACDF was performed at C4–C5. **b** MRI before revision surgery showing new degenerative changes at C3–C4 and C5–C6 causing stubborn upper limb pain and numbness. **c** Lateral radiograph after revision surgery showing that ACDF was performed at both the cranial and caudal levels. **d** MRI at 2 years postoperatively showing adequate decompression at the C3–C6 levels but new posterior disc herniation at C6–C7 (arrows). **e** Axial MRI at C6–C7 before and after the revision surgery showing aggravated disc herniation (arrows). The patient suffered from intermittent radiating pain in the right arm that was remediated by conservative treatment.

All surgical procedures were performed by the same senior surgeon. ACDF was carried out through a right-sided incision. The anterior cervical plates of the primary surgery were removed. Due to serious neural decompression, the osteophyte, posterior longitudinal ligament, and disc were completely excised. The endplates were prepared with a curette or burr. A PEEK cage filled with autogenous bone was then inserted and fixed using a locking plate. If a Zero-P cage was used, an additional plate was not applied (Fig. 1).

**Fig. 2:**
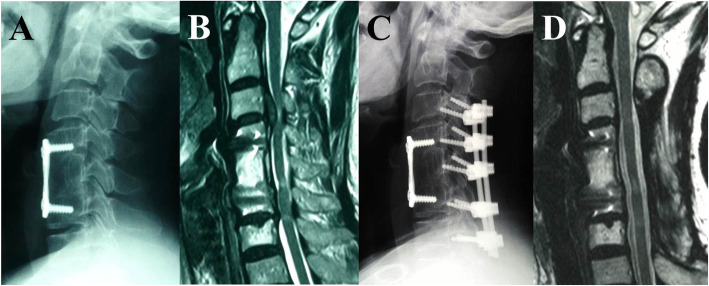
A 63-year-old woman developed two-level ASD with myelopathic symptoms at the 8th year after the primary surgery and underwent PDF with instrumented fusion as a revision surgery. **a** Lateral radiograph after the primary surgery showing that ACCF was performed at C5. **b** MRI before revision surgery showing a large disc extrusion behind the C3 vertebral body accompanied by ISI on the spinal cord at C3–C4 and C6–C7. **c** Lateral radiograph after revision surgery showing that posterior decompression with instrumented fusion was performed at C3–C7. **d** MRI at 2nd year postoperatively showing resorption of the herniated disc and release of spinal cord compression but with residual ISI at C6–C7.

PDF was carried out through a posterior midline incision, and the paravertebral muscles were retracted laterally. Lateral mass screws were placed bilaterally using the Magerl technique, and rods of appropriate size were selected. A laminectomy was then performed based on the preoperative MRI to obtain sufficient longitudinal decompression. The facet joints were decorticated, and a morselized local bone graft was packed into the facet joints and along the lateral masses (Fig. 2).

### Clinical and radiographic evaluation

Perioperative data such as fused levels, blood loss, length of hospitalization, and complications were recorded. Clinical and radiological evaluation data were collected preoperatively and at 3, 6, 12, and 24 months after surgery. When the follow-up was longer than 2 years, the last data available were used for statistical analysis. In this series, we only evaluated the preoperative and last follow-up data.

The modified Japanese Orthopedic Association (JOA) scoring system was used to determine the neurological status. The recovery rate (RR) was calculated as RR (%) = (Postoperative JOA score − Preoperative JOA score) / (17 − Preoperative JOA score) × 100%. The Neck Disability Index (NDI) score was used to assess the neck function. Visual analogue scale (VAS) scores were used to determine neck and arm pain. Radiographic evaluations included static and dynamic lateral images. Computed tomography and MRI scans were performed depending on the clinical status. Cervical lordosis was determined by the C2–C7 Cobb angle, formed by the angle between the inferior endplates of C2 and C7. If C7 was not visualized, C6 was utilized instead. Range of motion (ROM) of the cervical spine was measured as the C2–C7 Cobb angle on flexion/extension lateral radiographs. The number of recurrent ASD cases, defined as radiographic evidence of degeneration at the residual adjacent levels and associated relevant clinical symptoms occurring 6 months after the second operation, was observed.

### Statistical analysis

Statistical analyses were performed using SPSS version 16.0 software (SPSS Inc., Chicago, IL, USA). Data were presented as mean ± SD. Differences between preoperative and final follow-up measurements were analyzed by a paired-sample t test. An independent t test, the chi-square test, or Fisher’s exact text was used to identify significant differences between groups. Values of *p* < 0.05 were considered statistically significant.

## Results

### Perioperative characteristics

All patients completed a 2-year follow-up after the revision operation. The patient demographic characteristics including sex, time from primary arthrodesis, and follow-up duration did not differ significantly between the two groups (Table 2). Patients in the PDF group were more likely to be older than those in the ACDF group (*p* = 0.034). Regarding symptoms before the revision surgery, a higher proportion of patients presented with myelopathic symptoms in the PDF group (*p* = 0.027), while patients in the ACDF group had a higher rate of radicular symptoms (*p* = 0.012). Patients in the ACDF group had one level and three levels fused before and after the revision surgery, respectively. In the PDF group, 12 (85.7%) patients had two levels fused after the primary ACCF, and 4 or 5 levels were decompressed and fused in the second posterior operation. The ACDF group had a shorter mean surgery time (*p* < 0.001) and less blood loss than the PDF group (*p* < 0.001). The PDF group had a longer hospital stay than the ACDF group (*p* = 0.002).

**Table 2:** The perioperative characteristics between two groups

Variables	ACDF group	PDF group	*p* values
No. of patients	18	14	
Age (years)	52.6 ± 7.5	58.5 ± 7.4	0.034
Gender			
Male	10 (55.6%)	9 (64.3%)	0.725
Female	8 (44.4%)	5 (35.7%)	
Time from primary arthrodesis (years)	6.2 ± 2.4	6.8 ± 2.7	0.498
Surgery time (min)	117 ± 21	176 ± 28	< 0.001
Blood loss (ml)	186 ± 57	498 ± 176	< 0.001
Hospital stays (days)	8.6 ± 2.2	11.1 ± 2.0	0.002
Follow-up (months)	33.8 ± 6.9	36.1 ± 6.8	0.368
Symptoms before revision			
Myelopathy	3 (16.7%)	8 (57.1%)	0.012
Radiculopathy	11(61.1%)	2 (14.3%)	0.027
Myeloradiculopathy	4 (22.2%)	4 (28.6%)	0.703

### Clinical outcomes

The JOA, NDI, and both arm pain and neck pain VAS scores showed significant improvement compared with the preoperative scores in both groups (Table 3). Notably, patients in the PDF group had significantly lower preoperative JOA scores than those in the ACDF group (*p* = 0.030). However, there were no significant differences for postoperative JOA scores (*p* = 0.192) and RR values for neurological function (*p* = 0.787) at the final follow-up. Although the preoperative NDI scores were comparable between the two groups (*p* = 0.682), the NDI scores in the ACDF group were significantly superior than those in the PDF group at the final follow-up (*p* = 0.012). Although patients in the ACDF group had significantly higher preoperative arm pain VAS scores (*p* = 0.024), the postoperative arm VAS scores did not differ significantly between the two groups (*p* = 0.732). There was no significant difference in the preoperative neck pain VAS scores between the two groups (*p* = 0.535). Of note, patients who underwent PDF had significantly higher neck pain VAS scores at the final follow-up (*p* = 0.019).

**Table 3:** Clinical outcomes between two groups

Variables	ACDF group	PDF group	*p* values
No. of patients	18	14	
Pre JOA	11.1 ± 1.6	9.6 ± 1.8	0.030
Final FU JOA	14.3 ± 1.4^*^	13.6 ± 1.5^*^	0.192
RR (%)	57.7 ± 12.6	56.5 ± 12.1	0.787
Pre NDI	32.89 ± 8.07	34.07 ± 7.98	0.682
Final FU NDI	16.67 ± 5.09^*^	22.21 ± 6.61^*^	0.012
Pre arm VAS	6.3 ± 1.7	4.9 ± 1.6	0.024
Final FU arm VAS	2.4 ± 1.5^*^	2.6 ± 1.5^*^	0.732
Pre neck VAS	5.3 ± 1.7	5.7 ± 2.2	0.535
Final FU neck VAS	2.4 ± 1.5^*^	3.9 ± 2.0^*^	0.019

*Pre* pre-operative, *FU* follow-up

^*^p < 0.05, compared with the pre-operative data

### Radiographic outcomes

Although there was no significant difference in cervical lordosis between the two groups preoperatively (*p* = 0.619) (Table 4), it improved by 7.1° in the ACDF group and declined by 5.8° in the PDF group. So there was a significant difference in cervical lordosis between the two groups at the final follow-up (*p* = 0.002). Both groups exhibited significant postoperative decreases in cervical ROM compared with the preoperative values, but the ROM in the ACDF group was significantly greater than that in the PDF group at the final follow-up (*p* = 0.001).

**Table 4:** Radiographic outcomes and complications between two groups

Variables	ACDF group	PDF group	*p* values
No. of patients	18	14	
Pre lordosis	8.8° ± 5.5°	10.7° ± 5.0°	0.619
Final FU lordosis	15.9° ± 11.6°^*^	4.9° ± 6.4°^*^	0.002
Pre ROM	35.4° ± 9.7°	31.5° ± 8.9°	0.252
Final FU ROM	20.3° ± 8.6°^*^	9.1° ± 4.5°^*^	0.001
Complications	16.7%	21.4%	0.540
	Dysphagia (3, 16.7%)	C5 palsy (2, 14.3%)	
	-	Hematoma (1, 7.1%)	
Recurrent ASD	2 (11.1%)	0	0.308

*Pre* pre-operative, *FU* follow-up

^*^p < 0.05, compared with the pre-operative data

### Complications

In the ACDF group, 3 (16.7%) patients developed transient mild dysphagia after revision surgery, but this resolved spontaneously within 3 months. In the PDF group, 2 (14.3%) patients experienced C5 root palsy that was treated by active and passive shoulder ROM exercises, rest, and drug administration and showed full recovery. Postoperative hematoma occurred in 1 (7.1%) patient in the PDF group and was treated by emergency revision surgery. Recurrent ASD after the second operation occurred in 2 patients (11.1%) in the ACDF group (Fig. 1) but no patients in the PDF group. Both patients complained of recurrent and intermittent neck pain or radiculopathy, and MRI examination showed new posterior disc herniation at a residual adjacent segment. The patients were treated with conservative measures, such as cervical orthoses and physiotherapy combined with steroidal or nonsteroidal pharmacological agents, and a third cervical decompression surgery was not required at the final follow-up. There was no instrument failure or pseudoarthrosis during follow-up.

## Discussion

### Symptomatic ASD

In recent years, symptomatic ASD, a main concern after anterior cervical arthrodesis, has become a common challenge for surgeons. Although some experts still consider ASD to be a consequence of natural history, many reports attribute ASD to the compensatory increases in workload on the neighboring disc segments after vertebral arthrodesis [3, 15]. Surgical factors such as inadvertent intraoperative injury to an adjacent disc [16] and use of longer plate impinging [17] were also reported to be risk factors for ASD. Patients with acute or subacute neurological changes and invalid conservative treatment should be treated with further surgery. Revision surgery for symptomatic ASD was reported to be required in 5.1% and 22.2% of cases [3, 4, 7–9] and can be performed by second anterior fusion (ACDF or ACCF) [7, 10, 11], laminoplasty [12], posterior repeat cervical fusion [18], or artificial disc replacement [13, 14]. However, two-level ASD is a special type of ASD that is rarely reported. O'Neill et al. [11] retrospectively reviewed 40 patients who underwent ACDF for ASD and described that ASD occurred at both adjacent levels in 10% of cases. Lee et al. [8] analyzed 78 patients who required reoperation for ASD and found that 16 patients (20.5%) underwent treatment at both cephalad and caudal segments. Chen et al. [7] reported that 18 of 63 patients suffered from two-level ASD and underwent revision ACDF. To our knowledge, this is the first study to focus on revision surgical approaches and outcomes of two-level ASD. The study evaluated 32 patients who developed new neurological symptoms compatible with two lesions in the adjacent segments confirmed by MRI, including 10 patients who underwent their index surgeries at other hospitals. Thus, we were unable to calculate the true rate of revision surgery in our case series. However, this study aimed to compare outcomes of anterior versus posterior repeat surgery for this intractable pathologic condition.

### Choice of reoperation

The treatment for two-level symptomatic ASD followed the same principles used for patients with multilevel primary cervical spondylosis. The primary goal of the second surgery remained relief of neurological compression, stabilization of cervical spine, and restoration of lordotic alignment. In this study, the quality (soft or ossified) and position (disc level or retrovertebral) of compression, presenting symptoms, and pathological extent were the main factors considered during selection of the surgical approaches. Second anterior cervical fusion had been reported to achieve favorable clinical results in patients who underwent one-level ACDF for symptomatic ASD [7, 10, 11]. Furthermore, radicular symptoms could be better relieved by decompressing the nerve root in anterior surgery. However, patients with a narrow spinal canal at the primary operative segments or OPLL were not easily resolved by the anterior approach and required extensive decompression by a posterior approach [18–20]. Meanwhile, patients with reoperations at C2–C3 or C7–T1 may initially be considered for posterior surgery. In the present series, patients who underwent PDF had two levels fused after primary ACCF accompanied by two levels of adjacent symptomatic lesions. Therefore, posterior decompression with instrumented fusion is recommended because it can widely expand the cervical spinal canal and stabilize the motions of the adjacent segments. No patients underwent treatment with a combined anterior and posterior approach. Cervical disc arthroplasty (CDR) was reported to be effective for treatment of ASD [13, 14], but its indications were very strict, and long-term data were limited [14]. Implantation of a replacement disc adjacent to a prior fusion is likely to be more challenging than primary disc replacement. Therefore, CDR was not selected in the present study.

### Comparison of anterior and posterior reoperations

In the present study, patients who underwent PDF were older and had worse preoperative JOA scores than those who underwent ACDF because they had greater myelopathic preoperative deficits and more cases of multilevel initial fusion. Our data are consistent with the findings of Bydon et al. [18]. Patients who underwent PDF also experienced higher blood loss and longer hospitalizations, which may be caused by the increased number of decompressed and fused levels. In a cohort of patients who experienced pseudoarthrosis after ACDF, Carreon et al. [21] also reported greater surgical trauma after rearthrodesis in the posterior cohort compared with the anterior cohort. However, PDF was still a reasonable option that could achieve satisfactory neurological function for more extensive and effective decompression.

The VAS score for arm pain was significantly higher in the ACDF group preoperatively because a higher proportion of patients suffered from radiculopathy. However, there was no significant difference postoperatively because the nerve root can be better relieved by ACDF [7]. The patients in the PDF group experienced higher VAS neck pain scores after the second surgery, which may have been mainly caused by nuchal muscle intraoperative injury and facet joint destruction. Furthermore, the ROM of the cervical spine was significantly decreased by the extensive fusion in both groups. However, better ROM was retained in the ACDF group with relatively fewer fused levels. Better release and distraction were achieved by the anterior approach, but straightening of the cervical spine was obtained during the posterior approach, as previously reported [22, 23]. Owing to the better ROM and lordosis achieved by the anterior approach [20, 22], the NDI scores in the ACDF group were superior to those in the PDF group.

The overall complication rates were similar in the ACDF and PDF groups (16.7% vs. 21.4%), but dysphagia occurred at a higher rate in the ACDF group. Dysphagia has been reported to range from 4 to 30% [24], although certain measures, such as contralateral incision [25], preservation of previous cervical plates [7], limited exposure of responsible disc levels, and use of Zero-P cage [26], can decrease esophageal disturbance and retraction pressure. The posterior approach avoided extensive dissection through prevertebral scar tissue, but was more frequently associated with C5 palsy, postoperative hematoma, and axial pain. All of these complications were similar to those in the primary cervical surgery, but patients undergoing revision surgery for ASD were more likely to be discharged to a rehabilitation center rather than home.

### Recurrent ASD after revision surgery

Recurrent ASD after second cervical fusion, a serious long-term complication, was the greatest concern. In our study, two patients experienced recurrent neck pain or radicular symptoms in the ACDF group after the second operation, but a third decompression surgery was not required after the 2-year follow-up. Xu et al. [9] reported that patients who underwent a second anterior cervical fusion had a higher chance of developing recurrent ASD (29.9%) than patients who underwent a posterior reoperation (12.9%). In the study by Bydon et al. [18], the rate of requiring two revision surgeries in the anterior cohort was higher than that in the posterior cohort (32.5% vs. 16.1%). Repeated anterior fusion may result in further lesions in adjacent segments because it places more strain on the spinal biomechanics than the index ACDF itself [6]. Furthermore, patients with ASD have a propensity to develop degenerative changes in other non-fused levels. In contrast, posterior revision surgeries involved fusion of more levels and left the patients at less risk of developing recurrent ASD. However, the optimal mechanism warrants further investigation.

## Limitations

This study is the first to report on the revision approaches and clinical outcomes for treatment two-level symptomatic ASD. However, the main limitations of the study are related to its retrospective nature and limited cohort size. With increased numbers of ASD cases, prospective and randomized study designs will achieve higher degrees of evidence and the long-term results of repeat surgery warrant further evaluation.

## Conclusions

Both anterior and posterior decompression and fusion were effective procedures for improving the neurological outcomes of patients with two-level symptomatic ASD after anterior cervical arthrodesis. For patients with recurrent radiculopathy, second ACDF had less surgical trauma, better restoration of lordosis, and less postoperative neck pain, but a higher chance of ASD recurrence. For older patients with myelopathic symptoms, posterior revision surgery may be more reasonable, but long-term follow-up studies are still necessary.

## Data Availability

Data requests are available from the corresponding author.

## Abbreviations

ACDF: Anterior discectomy and fusion
ASD: Adjacent segment disease
CDR: Cervical disc arthroplasty
ISI: Increased signal intensity
JOA: Japanese Orthopedic Association
MRI: Magnetic resonance imaging
NDI: Neck Disability Index
PDF: Posterior decompression and fusion
ROM: Range of motion
VAS: Visual analog scale
